# Changes in Ageism and LGBTIA Clinical Competency Following an LGBTIA Older Adult Competency Training

**DOI:** 10.1093/geroni/igaf122.3728

**Published:** 2025-12-31

**Authors:** Jennifer Smith, Sharon Bowland, Jordan Westcott, Joseph Winberry

**Affiliations:** University of Tennessee, Knoxville, Knoxville, Tennessee, United States; University of Tennessee, Knoxville, Tennessee, United States; University of Tennessee at Knoxville, Knoxville, Tennessee, United States; UNC Chapel Hill, Chapel Hill, North Carolina, United States

## Abstract

Lesbian, gay, bisexual, transgender, intersex, agender/asexual, and other sexual and gender minority (LGBTIA) older adults face health disparities relative to the general population (Lampe et al., 2023), due in part to unmet health needs through available healthcare and aging services (Gibson et al., 2019). Lack of culturally competent services emerged in a community needs assessment as a concern among LGBTIA older adults (Winberry, 2023). To address this, we conducted an LGBTIA older adult cultural competency training focused on preparing healthcare and other service providers with the knowledge and skills necessary to meet the needs of this population. Using paired sample t-tests, we examined how the training impacted ageist attitudes and LGBTIA clinical competency among participants (n = 43). The training reduced ageist attitudes among participants, t(33) = -3.94, p < .001 with a moderate effect size (d = .47; Cohen, 1988). We also found that the training improved self-perceived clinical competency for working with LGBTQ+ individuals among participants, t(37) = 7.64, p < .001 with a moderate effect size (d = .58; Cohen, 1988). We found similar results for clinical preparedness, t(39) = 2.57, p = .014 with a moderate effect size (d = .53; Cohen, 1988); attitudes toward LGBTIA individuals, t(39) = 2.57, p = .014 with a small effect size (d = .22; Cohen, 1988); and knowledge about LGBTIA individuals, t(39) = 2.57, p = .014 with a moderate effect size, (d = .44; Cohen, 1988). Implications for interdisciplinary trainings focused on LGBTIA older adults will be discussed.

